# Boldenone and Testosterone Production from Phytosterol via One-Pot Cascade Biotransformations

**DOI:** 10.3390/jof10120830

**Published:** 2024-11-28

**Authors:** Vyacheslav V. Kollerov, Tatiana A. Timakova, Andrei A. Shutov, Marina V. Donova

**Affiliations:** Federal Research Center, Pushchino Center for Biological Research of Russian Academy of Sciences, G.K. Skryabin Institute of Biochemistry and Physiology of Microorganisms, Prospekt Nauki, 5, 142290 Pushchino, Moscow Region, Russia; tatianka.rz@yandex.ru (T.A.T.); w___w@rambler.ru (A.A.S.); mv_donova@rambler.ru (M.V.D.)

**Keywords:** phytosterol, *Curvularia*, cascade biotransformation, 17β-HSD, testosterone, 1-dehydrotestosterone, boldenone, 7α-hydroxylation

## Abstract

Testosterone (TS) and its 1(2)-dehydrogenated derivative boldenone (BD) are widely used in medicine, veterinary science and as precursors in organic synthesis of many therapeutic steroids. Green production of these compounds is possible from androstenedione (AD) enzymatically, or from phytosterol (PS) using fermentation stages. In this study, the ascomycete *Curvularia* sp. VKM F-3040 was shown to convert androstadienedione (ADD, 4 and 10 g/L) to yield 97% and 78% (mol/mol) of BD, respectively. Based on its high 17β-hydroxysteroid dehydrogenase (17β-HSD) activity, a novel cascade biotransformation of PS was developed for production of TS and BD. At the first stage, the strains of *Mycolicibacterium neoaurum* VKM Ac-1815D or *M. neoaurum* VKM Ac-1816D converted PS (5 or 10 g/L) into AD or ADD (each in a concentration of 2.5 or 5 g/L), respectively. At the second stage, mycelium of the fungus under the revealed optimal conditions reduced AD or ADD with more than 90% efficiency to form TS or BD, respectively. Based on transcriptome analysis, six candidate genes that might encode 17β-HSDs in the *Curvularia* sp. genome were revealed. Along with 17β-HSDs, the fungus possessed inducible P450_cur_ 7-monooxygenase, which led to the accumulation of 7α-hydroxytestosterone (7α-OH-TS) as a major product from AD (up to 83% within 24 h after mycelium addition at the second stage of cascade biotransformation). The presence of protein synthesis inhibitor cycloheximide (CHX) prevented 7α/β-hydroxylation due to inhibition of de novo synthesis of the enzyme in the fungal cells. The results demonstrate the high biotechnological potential of the *Curvularia* sp. strain and open up prospects for the synthesis of valuable 17β-reduced and 7-hydroxylated steroids by cascade biotransformations.

## 1. Introduction

Testosterone (TS) and its 1(2)-dehydrogenated analogue boldenone (BD) are valuable steroid compounds with a number of therapeutic properties and are widely used in medicine and veterinary science [1,2,3]. TS and BD play an important role in the human body, primarily in men, supporting reproductive function and influencing the prostate gland, hair growth, development and maintenance of muscle mass; BD, which has a more pronounced androgenic effect, is also used in the treatment of low TS levels [4,5,6].

**Figure 1:**
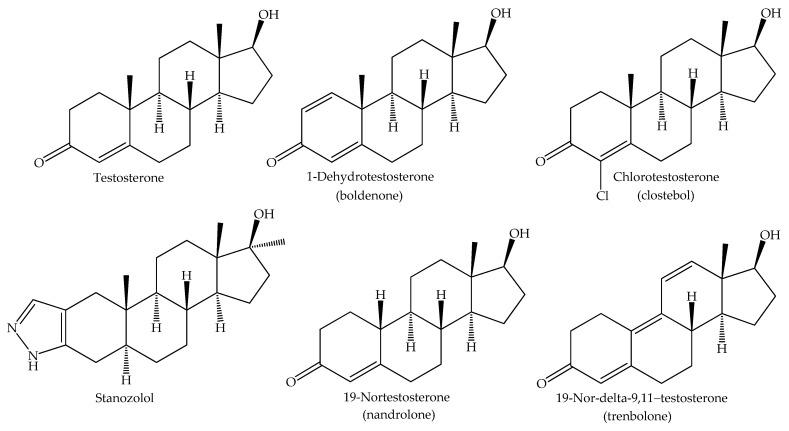
Examples of 17β-hydroxy-androstanes approved for veterinary use.

In some countries, TS or its synthetic derivatives possessing higher anabolic activity with minimal undesirable androgenic effects find application in animal production as growth stimulants in fattening or for dermatological use (Figure 1) [7,8,9,10]. On the other hand, BD is approved as a veterinary drug in many countries for the treatment of cattle, lambs and especially horses [1,11,12]. It has also been shown that BD, as an androgenic steroid, can improve growth and food conversion in food-producing animals [13]. Furthermore, BD is a long-acting drug, and the effects can persist for more than a month after intramuscular injection [14].

The main precursors of TS and BD synthesis are primary androstane steroids: androstenedione (AD) and androstadienedione (ADD), whose production is usually based on the ability of some wild and mutant strains of actinobacteria, mainly the *Mycolicibacterium* genus, to catalyze oxidative degradation of the aliphatic side chain of PS (a mixture of plant sterols, mainly C27 steroids) [15,16,17]. Further structural modification of AD and ADD to their high-demand 17β-reduced derivatives usually requires several stages of non-ecological chemical synthesis [18], or can be carried out enzymatically [19].

It should be noted that actinobacteria, including mycolicibacteria, usually possess their own 3(17)-hydroxysteroid dehydrogenases, whose activities can lead to the formation of TS as a minor product during the biotransformation of PS [15]. Recombinant strains of mycolicibacteria have been created expressing heterologous 17β-hydroxysteroid dehydrogenases (17β-HSD) and capable of TS production from PS, but none of them have been implemented in practical production so far [6,18,20,21,22]. Interestingly, in mycolicibacteria, 17β-reduction is accompanied by 1(2)-reduction, leading to the formation of TS as the main product from ADD [23].

Cascade biotransformations using different microorganisms in one bioreactor allow effective production of steroid 17β-alcohols (such as TS or BD) without the intermediate isolation of AD or ADD. For example, combination of PS bioconversion by *M. neoaurum* VKM Ac-1815D and C17β-carbonyl reduction by *Nocardioides simplex* VKM Ac-2033D in one bioreactor provided effective TS production [24]. Effective BD production was ensured by a microbial community of mutant *M. neoaurum* and the recombinant yeast *Pichia pastoris* [25].

Mold fungi of *Aspergillus*, *Acremonium*, *Doratomyces*, *Cochliobolus*, *Fusarium*, *Rhizopus*, *Nostoc*, *Pleospora*, *Trichoderma*, *Mucor* and *Pleurotus* genera were reported to catalyze 17-keto reduction of 3-oxo androstane steroids; however, in many cases 17β-HSD activity and the process’s efficiency were not high [26,27,28,29,30,31,32].

During a wide screening of filamentous fungi of different taxonomies for activity towards AD and ADD, we discovered a strain of ascomycete *Curvularia* (previously classified as *Drechslera*) sp. VKM F-3040 with the highest 17β-HSD activity [26]. Along with 17-keto reduction, the strain performed 7α/β-hydroxylation of steroids. Inhibitory analysis confirmed the constitutive character of 17β-HSD and inducible character of 7-hydroxylase in the strain [33].

In this study, this strain, in combination with AD- and ADD-producing mycolicibacteria strains, was applied in cascade biotransformations to produce valuable steroid 17β-alcohols (BD and TS) and their 7-hydroxylated derivatives from PS. Transformation of PS to AD by *Mycolicibacterium neoaurum* VKM-Ac-1815D was followed by AD to TS bioconversion using *Curvularia* sp. VKM F-3040. To produce BD, a combination of ADD-producing *M. neoaurum* VKM Ac-1816D with the fungus was applied. Prolonged biotransformation using *Curvularia* sp. allowed for the production of 7-hydroxylated 17β-hydroxysteroids of the androstane series in cascade bioreactions from PS.

## 2. Materials and Methods

### 2.1. Chemicals

Steroids: Phytosterol was obtained from Jiangsu Spring Fruit Biological Products Co., Ltd. (Taixing, China); androst-4-en-3,17-dione (AD), androst-1,4-diene-3,17-dione (ADD), androst-4-en-17β-ol-3-one (testosterone, TS) and androsta-1,4-diene-17β-ol-3-one (1-dehydrotestosterone, BD) were purchased from Sigma-Aldrich (Saint Louis, MO, USA). Methyl-β-cyclodextrin (MCD) was purchased from Wacker Chemie (München, Germany); bactoagar and yeast extract from Panreac (Barcelona, Spain); corn extract from Sigma-Aldrich (USA). Other reagents were of the best purity grade from domestic commercial suppliers.

### 2.2. Microorganisms and Cultivation

*Mycolicibacterium neoaurum* VKM Ac-1815D, *M. neoaurum* VKM Ac-1816D and *Curvularia* sp. VKM F-3040 were obtained from the All-Russian Collection of Microorganisms (VKM IBPM RAS).

#### 2.2.1. Cultivation of Mycolicibacteria

*Mycolicibacterium neoaurum* VKM Ac-1815D and VKM Ac-1816D strains were grown in two generation stages; incubation was carried out in 750 mL Erlenmeyer flasks (working volume 100 mL) under aerobic conditions on a rotary shaker (200 rpm) at 30 °C for 48 h as previously described [21].

#### 2.2.2. Cultivation of Fungal Strain

To obtain the first generation stage mycelium, a spore suspension of *Curvularia* sp. from one wort-agar slant was inoculated into 50 mL of a medium A containing (g/L): potato starch—45, yeast extract—10, corn steep—10 (pH 6.5), and incubated in a 750 mL Erlenmeyer flask aerobically on a rotary shaker (200 rpm) at 28 °C for 48 h. Medium B, containing (g/L): maltose—40, yeast extract—5, corn steep—5, K_2_HPO_4_—2, KH_2_PO_4_—16, MgSO_4_—0.5, FeSO_4_—0.05 (pH 6.5), was inoculated with an aliquot (10%, *v*/*v*) of the first generation mycelium and incubated for 24 h under the conditions described above. The grown mycelium was collected by centrifugation at 1800× *g* for 20 min and re-suspended in 0.05 M potassium phosphate buffer (pH 6.5) to a concentration of 20–60 g/L (in terms of dry weight) for steroid transformation.

### 2.3. Transformation of Steroids

#### 2.3.1. PS Bioconversion by Mycolicibacteria

For bioconversion of PS, 5% (*v*/*v*) of the second generation inoculum of *M. neoaurum* VKM Ac-1815D or *M. neoaurum* VKM Ac-1816D was transferred to a transformation medium (95 mL) containing (g/L): glucose—10, soy flour—20, corn extract—10, CaCO_3_—3, Tween 80—2.5, urea—0.25, with 0.02 M sodium–phosphate buffer (pH 7.0), methylated β-cyclodextrin (MCD) (0.8:1.0 molar ratio to PS) and PS (5 or 10 g/L) added before sterilization. Bioconversion was carried out in 750 mL Erlenmeyer flasks (working volume 100 mL) on a rotary shaker (200 rpm) at 30 °C for 96 h.

#### 2.3.2. ADD Conversion by *Curvularia* sp. Mycelium

Steroid substrate ADD (1–10 g/L) was added to the resting mycelium re-suspended in 50 mL of 0.05 M potassium phosphate buffer (pH 6.5) as a hot dimethyl sulfoxide (DMSO) suspension (the final DMSO concentration in the transformation buffer did not exceed 1% *v*/*v*). The protein synthesis inhibitor cycloheximide (CHX) was added to a final concentration of 0.06 mg/mL. Bioconversion was performed aerobically in 750 mL Erlenmeyer flasks (working volume 50 mL) on a rotary shaker (200 rpm) at 28 °C for 1–72 h.

#### 2.3.3. Cascade Biotransformation

##### PS Conversion to TS

Culture broth obtained after the first stage of PS transformation by *M. neoaurum* VKM Ac-1815D (96 h) and containing AD as a major product was heated to 60 °C for 30 min to stop the vital activity and any steroid-transforming activity of bacterial cells. Then, the resting *Curvularia* sp. mycelium (60 g/L, by dry weight) was added and incubation was performed aerobically on a rotary shaker (200 rpm) at 28 °C for 1–72 h. In some experiments, CHX was added to a final concentration of 0.06 mg/mL.

##### PS Conversion to BD

Culture broth obtained after PS bioconversion using *M. neoaurum* VKM Ac-1816D (96 h) and containing ADD as a major product was heated to 60 °C for 30 min. Then, the resting *Curvularia* sp. mycelium was added and biotransformation was carried out as described above (in Section 2.3.1). In some experiments, CHX was added to a final concentration of 0.06 mg/mL.

### 2.4. Isolation of Steroids

Steroids were extracted from the culture broth (50–100 mL) with two volumes of ethyl acetate (EtOAc), which were then evaporated under vacuum to obtain crude residues. Individual steroid compounds were isolated using preparative column chromatography as described earlier [33].

### 2.5. Thin Layer Chromatography (TLC)

Cultivation broth samples (1–2 mL) were extracted with 2–4 mL of EtOAc. The extracts were applied to ALUGRAM SIL G/UV254 TLC sheets (Düren, Germany). A mixture of benzene–acetone (3:1.5, *v*/*v*) was used as mobile phase. UV detection at 254 nm on a CN-15MC UV Darkroom instrument (Vilber Lourmat, Marne-la-Vallée, France) was used to visualize 3-keto-4-ene steroids. Detection of 3β-ol-5-ene steroid derivatives was performed by staining of TLC-sheets with MnCl_2_ reagent and heating to 100–105 °C until color appeared.

### 2.6. High Performance Liquid Chromatography (HPLC)

Samples for the HPLC analyses were prepared as follows: aliquots (100–200 µL) of culture broth (each sample in triplicate) were diluted 20–25 times with an acetonitrile–isopropanol solution (50:45, *v*/*v*). The suspension was centrifuged at 12,100× *g* for 10 min. Aliquots of the supernatant were used for analyses.

The analyses were performed on an Agilent 1200 instrument (Agilent Technologies, Waldbronn, Germany) and a Symmetry C18 column (5 µm, 4.6 × 250 mm) with a Symmetry C18 precolumn (5 µm, 3.9 × 20 mm) (Waters, Milford, MA, USA). Calibrations were performed by an external standard method based on the comparison of peak areas. Processing of the results was carried out using ChemStation Rev. B. 04.03 (Agilent Technologies, Santa Clara, CA, USA).

The study was carried out under the following conditions: flow rate of 1 mL/min, column temperature 50 °C, detection of 3β-ol-5-en/3-keto-4-en configurations at 200/240 nm. Mobile phase for phytosterol: acetonitrile:isopropanol:water (50:45:5 *v*/*v*/*v*); for steroid products: acetonitrile:H_2_O:acetic acid (60:40:0.01 *v*/*v*/*v*). Retention time (R_t_): PS, 11.5 min; 20-HMP, 11.3 min; 20-HMPD, 8.15 min; AD, 6.1; TS, 5.2; ADD, 4.95; BD, 4.34 (Figures S1–S3).

To detect 7-hydroxylated derivativies of TS and BD, acetonitrile:H_2_O:acetic acid (52:48:0.01, *v*/*v*/*v*) was used as a mobile phase. Retention time (R_t_): 7β-OH-TS, 3.55 min; 7α-OH-TS, 3.22 min; 7β-OH-BD, 3.28 min; 7α-OH-BD, 3.01 min.

### 2.7. Mass Spectrometry (MS), ^1^H- and ^13^C-NMR Spectroscopy

MS spectra were recorded with a Bruker Maxis Impact spectrometer (Figure S4).

^1^H- and ^13^C-NMR spectra were recorded at 400 and 100.6 MHz, respectively, with a Bruker Avance 400 spectrometer. Chemical shifts were measured relative to a solvent signal (Figure S5).

The structure of steroid metabolites formed by *Curvularia* sp., including TS, BD, 7α-OH-TS, 7β-OH-TS, 7α-OH-BD and 7β-OH-BD, were defined based on the identification of the spectral data with those previously obtained for the corresponding compounds [33].

### 2.8. Quantification of Steroid Transformation Products

Conversion rate (CR) was estimated in % (mol/mol) based on HPLC data using the following equation:

$$CR=\frac{\left(mP/MP\right)}{\left(mS/MS\right)}\times 100\%,$$

where mP and mS are the weights of steroid products and steroid substrates, respectively; MS and MP are the molar weights of steroid substrate and product, respectively.

### 2.9. Statistical Data Processing

All experiments and measurements were carried out in triplicate. The standard deviations in the graphs for each sample are shown as error bars.

## 3. Results and Discussion

### 3.1. C17β-Carbonyl Group Reduction of ADD by Curvularia sp.

The presence of both constitutive 17β-HSD and inducible 7-hydroxylase activity in *Curvularia* sp. mycelium was confirmed in our previous study [33]. In this regard, the optimization of ADD 17-keto reduction to BD was carried out by the resting mycelium of the second generation (24 h), which showed high 17β-HSD activity of constitutive dehydrogenase in the absence of inducible 7-hydroxylase. To avoid 7-monooxygenase synthesis de novo in the fungal cells in response to a steroid substrate, the inhibitor of cytoplasmic protein synthesis cycloheximide (CHX) was added.

There are several examples demonstrating successful use of washed resting mycelia for bioconversion of steroid substrates, such as: 11α-hydroxylation catalysis by *Aspergillus ochraceus* [34]; 7α/β and 15α-hydroxylation of dehydroepiandrosterone (DHEA) by *Botryodiplodia malorum* and *Colletotrichum lini* [35]; as well as 11β- and 14α-hydroxylation of pregnane and androstane steroids catalyzed by different strains of *Curvularia lunata* (*Cochliobolus lunatus*): VKM F-644, VKPM F-981, VKPM F-988 [36,37,38,39].

**Figure 2:**
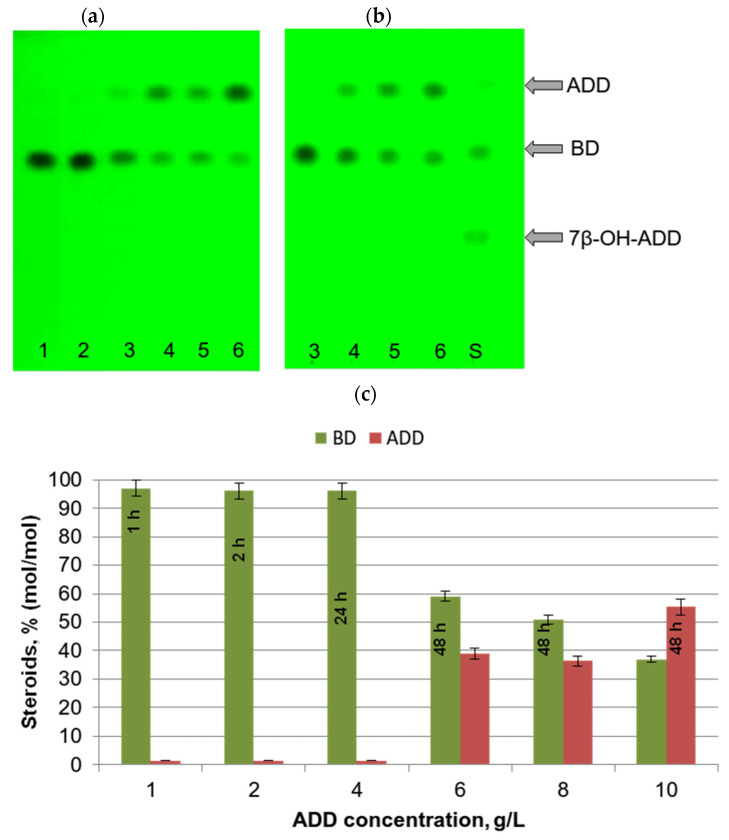
TLC profile of ADD transformation ((**a**)—6 h, (**b**)—48 h) by resting *Curvularia* sp. mycelium in the presence of protein synthesis inhibitor cycloheximide CHX (0.05 mg/mL); steroid substrate loads: 1 g/L (variant 1); 2 g/L (variant 2); 4 g/L (variant 3); 6 g/L (variant 4); 8 g/L (variant 5); 10 g/L (variant 6); S—a mixture of reference steroids in the order (from top to bottom): androstenedione (ADD); boldenone (BD); 7β-hydroxyandrostenedione (7β-OH-ADD). (**c**) Maximum BD yield detected in the culture broth samples at various intervals (1–48 h) while resting *Curvularia* sp. mycelium were incubated with ADD loaded in different concentrations.

When *Curvularia* sp. mycelium were incubated with ADD (1–4 g/L), BD was formed as the only product (Figure 2a,b, variants 1–3; Figure 2c). About 97% ADD (1–2 g/L) was converted to BD for 2 h only (Figure 2c, variants 1, 2), whereas full conversion of 4 g/L ADD completed within 24 h (Figure 2a,b, variant 3; Figure 2c). Further increases in ADD load (6–10 g/L) negatively affected ADD conversion. Thus, 40–60% of ADD remained unconverted even after 48 h of incubation, while the yield of BD ranged from 38 to 59% (mol/mol) (Figure 2a,b, variants 4–6; Figure 2c).

Probably due to extremely low steroid substrate solubility (less than 0.1 g/L), the increase in ADD concentration of up to 5–10 g/L in the transformation medium hindered accessibility of steroid molecules to the action of *Curvularia* sp. enzymes. The possible toxic effect of high steroid substrate concentrations on the viability of fungal mycelium should also be taken into account. The negative effect of high steroid substrate concentrations on transformation catalyzed by mold fungi was previously shown for different ascomycete and zygomycete strains. For instance, *Backusella lamprospora* mycelium actively catalyzed the 7α-hydroxylation of 1 g/L dehydroepiadrosterone (DHEA) producing over 50% 7α-OH-DHEA, whereas an almost twofold decrease in the target product yield was observed while increasing the steroid substrate concentration to 2–5 g/L [40]. The yield of an 11α-hydroxylated derivative dropped from 50 to 30% when pregnenolone concentration was increased from 0.5 to 1 g/L during incubation with *Aspergillus niger* [41]. Transformation of DHEA (0.5 g/L) by *Beauveria bassiana* provided an accumulation of up to 36% 11α-OH-DHEA, while an increase in steroid substrate load to 2.5 g/L was reflected in a threefold decrease in the yield of the target hydroxylated derivative [42]. A dramatic decrease in 11β-hydroxylated product yield (from 85–87% to 30–45%) by *Cunninghamella blakesleeana* and *Curvularia lunata* strains was observed when raising the steroid substrate (cortexolone acetate) load from 0.15 to 1 g/L and from 1 to 2 g/L, respectively [37,43].

**Figure 3:**
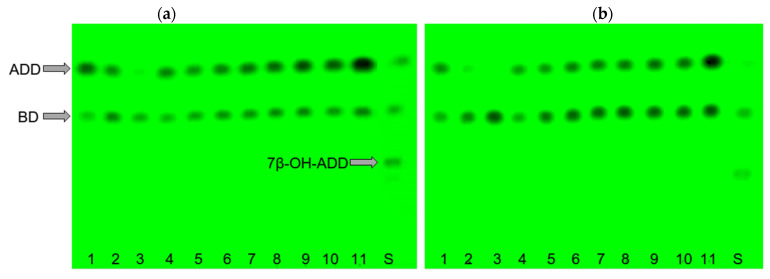
TLC profile of ADD (10 g/L) bioconversion in the presence of CHX (0.05 mg/mL) ((**a**)—24 h, (**b**)—48 h) by resting *Curvularia* sp. mycelium loaded to 20 g/L (variant 1), 40 g/L (variant 2), 60 g/L (variant 3), or 80 g/L (variant 4) without methylated β-cyclodextrin (MCD) addition (variant 1) or in the presence of MCD in a molar ratio of ADD 0.2:1 (variant 5), 0.5:1 (variant 6), 0.7:1 (variant 7), 1.2:1 (variant 8), 1.5:1 (variant 9), 1.7:1 (variant 10), or 2:1 (variant 11); S—a mixture of reference steroids in the order (from top to bottom): ADD, BD; 7β-OH-ADD.

**Figure 4:**
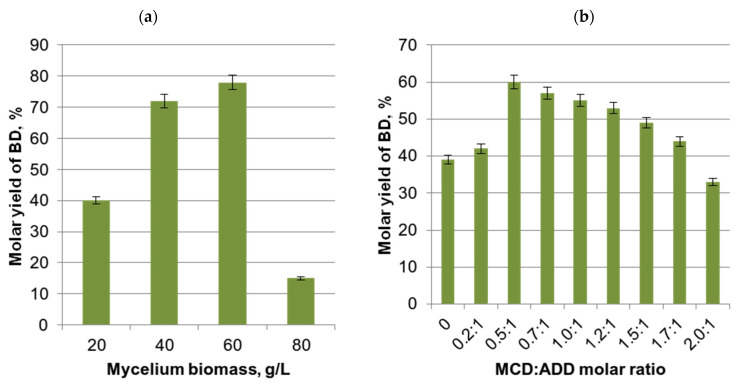
Influence of fungal biomass (**a**) and various concentrations of MCD (**b**) on ADD (10 g/L) 17-keto reduction catalyzed by resting *Curvularia* sp. mycelium.

To increase the enzyme pool of the constitutive 17β-HSDs in *Curvularia* sp. mycelium, the effect of fungal biomass on the 17-keto reduction rate has been studied. A strong positive effect on ADD (10 g/L) conversion with an almost twofold increase in BD yield (to 78%) was observed when increasing the mycelium biomass to 60 g/L (Figure 3, variant 3; Figure 4a), although its further increase to 80 g/L hindered ADD conversion with a drop in BD yield to 15% (Figure 4). Probably, a denser biomass may be the reason for reduced oxygen transport to fungal cells, as was previously suggested when studying DHEA hydroxylation by *Colletotrichum lini* [44].

Earlier, when studying the stepwise biotransformation of PS using *Mycolicibacterium neoaurum* and *Pichia pastoris*, which heterologously express the 17β-HSD gene, higher BD productivity (up to 65%) was also observed while increasing the yeast cell biomass to 175 g/L, although its further increase had a negative effect on BD production [25]. The biomass of recombinant *M. neoaurum* strain MNR M3M-ayr1&g6p also greatly affected the production of BD from PS: the maximum yield of 17β-alcohol was observed when 70 g/L wet cells were used in the system, but did not increase further when the biomass was over 70 g/L [20].

It is known that chemically modified β-cyclodextrins increase steroid solubility and facilitate their transport into fungal cells by increasing the permeability of cell membranes [45,46], as well as providing more complete isolation of steroid products from the culture broth by precipitation of insoluble β-CD complexes [47].

In the present study, the influence of different concentrations of methylated β-cyclodextrin (MCD) on the efficiency of ADD (10 g/L) bioconversion into BD by the resting *Curvularia* sp. mycelium was studied. Solubilizing agent was used in a molar ratio to the steroid substrate ranging from 0.2 to 2 (Figure 3, variants 5–11; Figure 4). MCD addition enhanced the 17-keto reduction of ADD with maximum BD yield observed at the MCD/ADD molar ratio of 0.5:1 (Figure 3, variant 6; Figure 4b), which was 18–20% higher than that in the control without MCD addition (Figure 3, variant 1; Figure 4b).

Cyclodextrin-mediated enhancement of steroid bioconversions is well-documented for different microbial processes, including oxidative degradation of the side chain of PS or 1(2)-dehydrogenation of androstane steroid catalysis by actinobacteria, as well as regio- and stereospecific hydroxylation performed by filamentous fungi. For example, the addition of hydroxypropyl-β-cyclodextrin enhanced production of AD and ADD from PS by *M. neoaurum* strains [48,49,50], and contributed to a steroid 1-dehydrogenation reaction catalyzed by *Arthrobacter simplex* [51]. Increased production of 17α-hydroxyprogesterone from progesterone by *Curvularia lunata* ATCC 12017 was observed when using the method of complexation with hydroxypropyl-β-cyclodextrin during biotransformation [52]. The use of MCD in an equimolar ratio to DHEA provided a twofold increase in the production of 7α-OH-DHEA by the fungus *Backusella lamprospora* [40], contributed to a more complete transformation of the steroid substrate by *Colletotrichum lini* ST1 [53], and also provided the maximum yield of 7-hydroxy derivatives during bioconversion of DHEA with the recombinant yeast *Saccharomyces cerevisiae* carrying the 7α-hydroxylase (CYP7B1) gene [54].

**Scheme 1:**
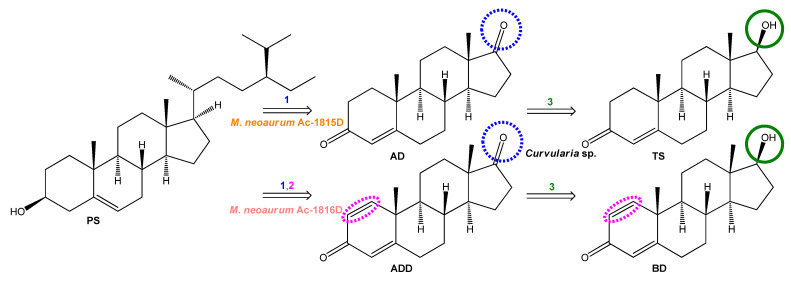
Cascade biotransformation of PS into TS or BD: 1, oxidative degradation of the side chain of PS; 2, 1(2)-dehydrogenation; 3, 17-keto reduction.

To summarize, the optimal conditions providing effective 17-keto reduction of ADD by the resting *Curvularia* sp. mycelium include the use of 60 g/L of fungal biomass (by dry weight) with the addition of MCD in a molar ratio to the steroid substrate of 0.5:1. The revealed conditions were further applied while developing cascade biotransformations of PS into TS or BD based on the use of AD or ADD-producing mycolicibacteria strains, respectively (first stage), and their subsequent 17-keto reduction by *Curvularia* sp. mycelium (second stage) (Scheme 1).

### 3.2. Cascade Biotransformation of PS into TS or BD

#### 3.2.1. Bioconversion of PS into AD or ADD (First Stage)

**Figure 5:**
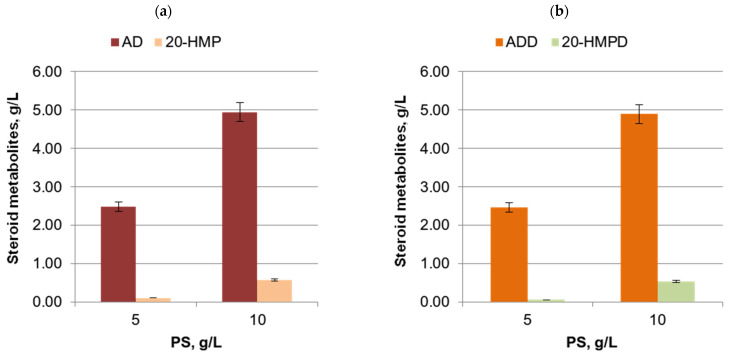
First stage of PS (5 or 10 g/L) bioconversion (96 h) by *M. neoaurum* VKM Ac-1815D (**a**) and *M. neoaurum* VKM Ac-1816D (**b**). Androstenedione (AD) and 22-hydroxy-23,24-bisnorchol-4-ene-3-one (HBC or 20-HMP) were formed by *M. neoaurum* VKM Ac-1815D (**a**). Androstadienedione (ADD) and 1(2)-dehydro-22-hydroxy-23,24-bisnorchol-4-ene-3-one (20-HMPD) were formed by *M. neoaurum* VKM Ac-1816D (**b**).

Incubation of *M. neoaurum* VKM Ac-1815D with PS (5 or 10 g/L) for 96 h resulted in the formation of 2.48 or 4.95 g/L of AD, respectively, whereas PS (5 or 10 g/L) transformation by *M. neoaurum* VKM Ac-1816D provided the accumulation of 2.47 or 4.9 g/L of ADD (Figure 5). 22-Hydroxy-23,24-bisnorchol-4-ene-3-one (HBC or 20-HMP) or its 1(2)-dehydrogenated analog (20-HMPD) were detected as the main byproducts; however, the yield of C-22 steroid metabolites did not exceed 10–12% (~0.5 g/L) at a PS load of 10 g/L (Figure 5).

#### 3.2.2. C17-Carbonyl Group Reduction of AD or ADD (Second Stage)

##### Obtaining of TS and 7a-OH-TS

**Figure 6:**
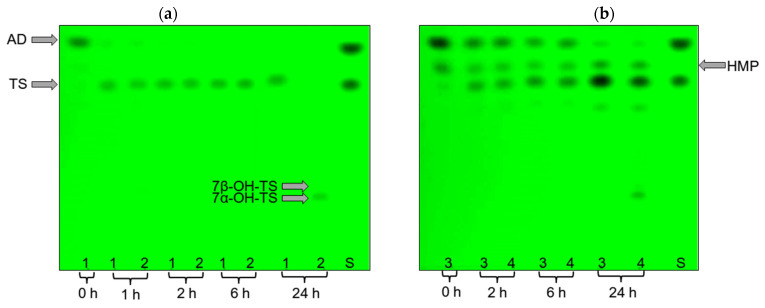
TLC chromatograms of culture broth samples during incubation with *Curvularia* sp. mycelium: (**a**)—culture broth containing 2.48 g/L AD (variants 1, 2); (**b**)—culture broth containing 4.95 g/L AD (variants 3, 4); variants 1, 3—with CHX addition; variants 2, 4—without CHX addition; S—reference steroids in the order (from top to bottom): AD, TS.

**Figure 7:**
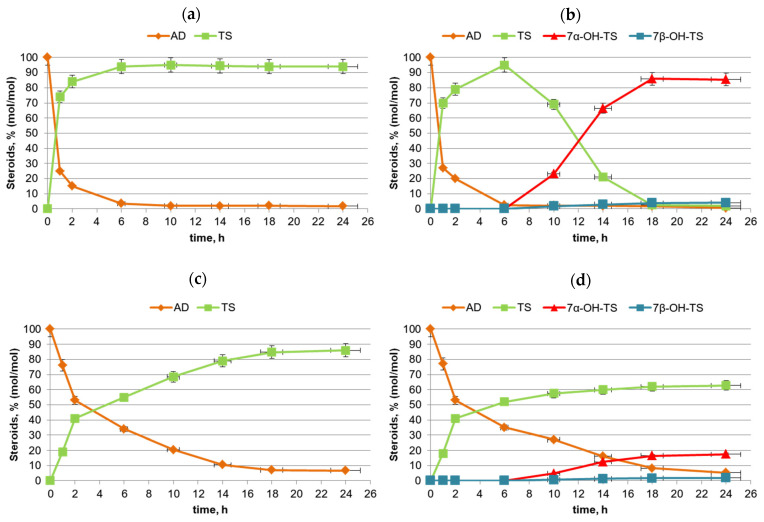
Time course of AD bioconversion by *Curvularia* sp. resting mycelium in culture broth obtained at the first stage of PS cascade biotransformation by *M. neoaurum* VKM Ac-1815D: (**a**,**b**)—culture broth containing 2.48 g/L (8.67 mM) of AD; (**c**,**d**)—culture broth containing 4.95 g/L (17.3 mM) of AD; (**a**,**c**)—with the addition of the protein synthesis inhibitor cycloheximide (CHX) 0.06 mg/mL; (**b**,**d**)—without CHX addition; in variants (**b**,**d**) 7α-hydroxytestosterone (7α-OH-TS) and 7β-hydroxytestosterone (7β-OH-TS) were also accumulated.

After PS to AD bioconversion by *M. neoaurum* VKM Ac-1815D, the fungal biomass was added to the culture broth. Almost full conversion of AD (2.48 g/L) into TS (more than 90%) was observed after 6 h incubation both in the presence of and without added CHX (Figure 6a, variants 1 and 2; Figure 7a,b).

Significant changes in the ratio of bioconversion products were observed after 24 h of incubation: TS was detected as a main product (90–92%) in the variant with added CHX (Figure 6a, variant 1; Figure 7a), whereas in the variant without added CHX, the entire amount of TS accumulated in the first hours of bioconversion was hydroxylated (mainly at the C7-Hα position) with accumulation of 7α-OH-TS as a major product (≥83%) and 7β-OH-TS as a minor metabolite (<4%), while the amount of unconverted TS did not exceed 0.7% (Figure 6a, variant 2; Figure 7b). Apparently, the cells of the resting mycelium synthesized the inducible steroid 7-hydroxylase (P450_cur_), which actively catalyzed the 7-hydroxylation of TS with its complete conversion into 7α/β-hydroxylated derivatives (Figure 6a, variant 2; Figure 7b). When inhibiting the de novo synthesis of the inducible 7-monooxygenase in fungal cells with CHX, no 7-hydroxylation of TS was observed (Figure 6a, variant 1; Figure 7a).

Previously, the ability to catalyze 7α-hydroxylation of TS was shown in the fungal strain *Botrytis cinerea*; however, the selectivity of the reaction was low [55]. The high regio- and stereospecificity of the 7α-hydroxylation reaction revealed in the present study for *Curvularia* sp. mycelium is of great interest, since 7α-hydroxylated derivatives of androstane steroids can be used as key precursors in the synthesis of pharmaceuticals with diuretic, anti-inflammatory and neuroprotective properties [55,56].

The rate of C17-carbonyl group reduction noticeably decreased when AD content in the culture broth obtained after the first stage of PS cascade biotransformation increased twofold: after 6 h of incubation 35–38% of the AD remained unconverted (Figure 6b, variants 3,4; Figure 7c,d), whereas its almost complete conversion into TS (more than 90%) was observed within the same incubation time when initial AD content was 2.48 g/L (Figure 6a, variants 1, 2; Figure 7a,b). Even after 24 h of incubation with mycelium, about 7% of the AD was still detected and the yield of TS did not exceed 83% (Figure 7c). A similar effect was observed when incubating the resting mycelium of *Curvularia lunata* VKPM F981 with dehydroepiandrosterone acetate (ADEA): full conversion into 7α-hydroxylated derivative occurred at an ADEA load of 2 g/L, whereas an increase in ADEA concentration in the reaction mixture to 5 g/L inhibited steroid transformation [40].

##### Obtaining of BD and Its 7a-Hydroxylated Derivative

**Figure 8:**
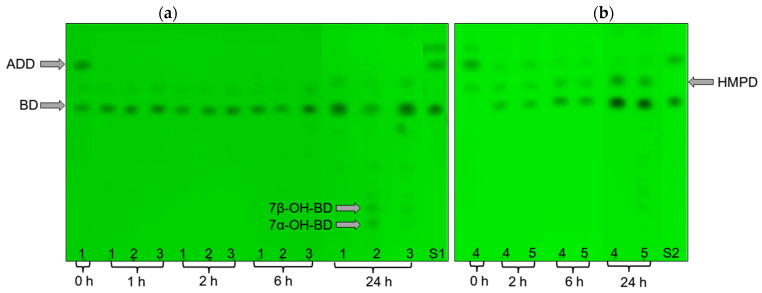
TLC chromatograms of culture broth samples during incubation with *Curvularia* sp. mycelium: (**a**)—culture broth containing 2.47 g/L ADD (variants 1, 2, 3); (**b**)—culture broth containing 4.9 g/L ADD (variants 4, 5); variants 1, 4—with CHX addition; variants 2, 3, 5—without CHX addition; variant 3—with MCD addition (0.5:1 molar ratio to ADD); S1—reference steroids in the order (from top to bottom): AD, ADD, BD; S2—ADD, BD.

**Figure 9:**
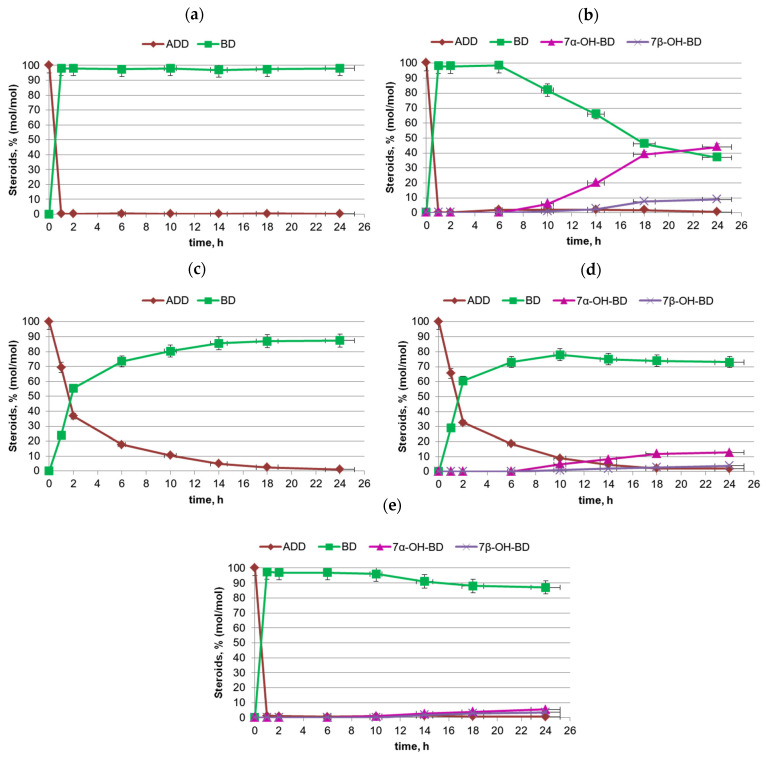
Time course of ADD bioconversion by *Curvularia* sp. resting mycelium in the culture broth obtained at the first stage of PS cascade biotransformation by *M. neoaurum* VKM Ac-1816D: (**a**,**b**,**e**)—culture broth containing 2.47 g/L (8.64 mM) of ADD; (**c**,**d**)—culture broth containing 4.9 g/L (17.25 mM) of ADD; (**a**,**c**)—with CHX addition; (**b**,**d**,**e**)—without CHX addition. Medium supplemented with methylated β-cyclodextrin (MCD) in molar ratio to steroid substrate as 0.5:1 (**e**); in variants (**b**,**d**,**e**), 7α-hydroxyboldenone (7α-OH-BD) and 7β-hydroxyboldenone (7β-OH-BD) were accumulated.

When the culture broth obtained after PS conversion to ADD by *M. neoaurum* VKM Ac-1816D was incubated with *Curvularia* sp. mycelium, ADD (2.47 g/L) almost completely reduced to BD in just 1 h (Figure 8a, variants 1–3; Figure 9a,b,e). Apparently, the presence of a 1(2)-double bond in ADD’s structure makes this compound a preferable substrate for the action of the fungal 17β-HSD enzyme. Such a high rate of reduction of the 17-carbonyl group of ADD indicates the high biotechnological potential of *Curvularia* sp. 17β-HSD, especially for the production of BD, which is in demand in veterinary medicine. 

When incubating for a longer time (2–6 h), the concentration of BD did not significantly change (Figure 8a, variants 1–3; Figure 9a,b,e). Nevertheless, after 24 h of transformation, partial conversion of BD into 7-hydroxylated derivatives, 7α-OH-BD (43.94%) and 7β-OH-BD (9.29%), was observed in the variant without added CHX (Figure 8a, variant 2; Figure 9b), while in the variant with added CHX the amount of BD did not change (Figure 8a, variant 1; Figure 9a).

It is noteworthy that the mycelium catalyzed the 7α/β-hydroxylation of BD less actively compared to TS, which was completely transformed into 7-hydroxyderivatives within 24 h (Figure 8a, variant 2; Figure 9b). Probably, TS is a more convenient substrate for the fungal steroid 7-hydroxylase than BD, with an unsaturated C1-C2 double bond in ring A, which could be the reason for the weaker binding of the steroid molecule to the catalytic center of P450_cur_ 7-monooxygenase.

Interestingly, the presence of MCD in the transformation medium prevented further structural modification (primarily 7-hydroxylation) of accumulated BD by *Curvularia* sp. mycelium: the total yield of 7α/β-hydroxylated derivatives of BD did not exceed 10% even after 24 h of incubation (Figure 8a, variant 3; Figure 9e).

When ADD content in the culture broth was 4.9 g/L, a lower rate of C17-keto reduction was observed: after 6 h of incubation with fungal mycelium, up to 15–18% of the ADD remained unconverted (Figure 8b, variants 4, 5; Figure 9c,d). Nevertheless, after 24 h incubation almost complete ADD-to-BD conversion was achieved (Figure 9d).

It should be noted that C-22 steroid metabolites (20-HMP or 20-HMPD) present in the culture broths after the first stage of PS (10 g/L) bioconversion with *M. neoaurum* VKM Ac-1815D or *M. neoaurum* VKM Ac-1815D, respectively, were not structurally modified by the fungal mycelium at the second stage and their quantity remained unchanged during the 24 h of incubation (Figure 6b, variants 3, 4; Figure 8b, variants 4, 5). This, in turn, indicates the absence of steroid-transforming activity in the fungal cells towards 20-HMP or 20-HMPD.

Successful use of mold fungi in the two-step synthesis of valuable steroid metabolites from PS was already demonstrated in our previous work: the fungal strain *Aspergillus ochraceus* VKM F-830 selectively catalyzed the 11α–hydroxylation of AD (the product of β-sitosterol bioconversion with *M. neoaurum* VKM Ac-1815D), providing the accumulation of 11α-OH-AD, which is a key precursor in the synthesis of glucocorticoids [57].

The results obtained in the present study demonstrate effective cascade biotransformation of PS to high-value TS and BD with a number of advantages over similar microbial processes, which are usually characterized by longer fermentation cycles as well as more difficult bioprocess implementation [22,23,24]. For instance, cascade bioconversion of PS into TS by *M. neoaurum* VKM Ac-1815D and *Nocardioides simplex* VKM Ac-2033D was complicated by a reversible redox reaction catalyzed by 17β-HSD of *N. simplex*: biotransformation of AD to TS actively proceeded only in the first hours of incubation and provided up to 41% of TS in 8 h, after which the reverse oxidation of TS to AD was observed [24]. It is worth noting the absence of such a reverse reaction of 17β-OH group oxidation while using *Curvularia* sp. mycelium, which can be considered an obvious advantage in the production of TS and BD from cheap and available raw plant materials.

### 3.3. Search for Candidate 17β-HSD Genes in Curvularia sp. Cells

**Figure 10:**
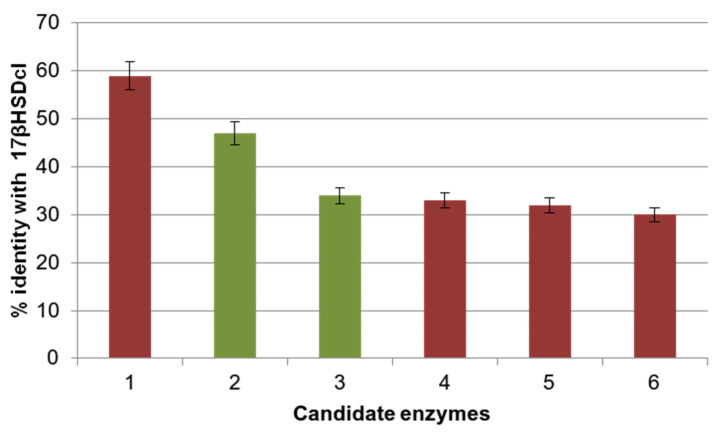
Putative 17β-HSD genes revealed in *Curvularia* sp. VKM F-3040: 17HSDcl nucleotide sequence from *C. lunata* (GenBank: AF069518) was used as a reference while sequence homology searching: short-chain dehydrogenases/reductases (SDR) (brown columns); Rossmann-fold domain-containing proteins (green columns).

In order to identify genes that might encode 17β-HSD in *Curvularia* sp. cells, the previously assembled de novo fungal transcriptome was used [58]. A 17β-HSDcl coding sequence from *Curvularia lunata* (GenBank: AF069518) was used as query while searching for candidate genes among the assembled transcripts of *Curvularia* sp. As a result, six genes with 28–57% homology with the reference 17HSDcl nucleotide sequence were revealed. The putative amino acid sequences were then used as queries for functional annotation by using the NCBI Conserved Domain Database (Figure 10). Four candidate genes were shown to encode short-chain dehydrogenases/reductases (SDRs) belonging to the NAD(P)-dependent oxidoreductase family, whereas two other genes encode Rossmann domain-containing NAD(P)-binding proteins capable of functioning as oxidoreductases (Figure 10).

Notably, the candidate SDR enzyme showing the maximum homology (57%) with 17HSDcl has a high amino acid sequence identity (95.9%) with trihydroxynaphthalene reductase (3HNR) from *Curvularia lunata* (MW659861.1) (protein BLAST data).

Analysis of differential expression revealed a comparable expression level of the six selected candidate 17β-HSD genes in both the control and the steroid-induced *Curvularia* sp. mycelium, thus confirming the constitutive character of the corresponding enzymes. Future work will be focused on functional identification of the revealed candidate genes via cloning and heterologous expression.

## 4. Conclusions

In the present study, the conditions providing maximum 17-keto reduction activity of the resting *Curvularia* sp. VKM F-3040 mycelium were defined. Based on the biocatalytic activities of the *M. neoaurum* VKM Ac-1815D and VKM Ac-1816D strains towards PS with formation of primary androstane steroids AD or ADD, and the ability of the resting *Curvularia* sp. mycelium to reduce the 17-keto group of the formed androstanes, novel effective cascade biotransformations of PS into TS or BD were developed. Additionally, the revealed conditions provided the accumulation of 7α-OH-TS as the main product of cascade PS transformation. Six candidate 17β-HSD genes were identified in the *Curvularia* sp. mycelium based on transcriptome analysis.

The results demonstrate high biotechnological potential of the developed cascade synthesis of valuable C17-reduced and 7-hydroxylated steroids from cheap and available plant sterols and open up the prospects for the creation of advanced green technology demanded by the pharmaceutical industry.

## Data Availability

The original contributions presented in the study are included in the article and Supplementary Materials, further inquiries can be directed to the corresponding authors.

## Supplementary Materials

The following supporting information can be downloaded at: https://www.mdpi.com/article/10.3390/jof10120830/s1, Figure S1: HPLC calibration curves of standard reference of androstenedione (AD) (A); androstadienedione (ADD) (B); testosterone (T) (C), boldenone (BD) (D); 22-hydroxy-23,24-bisnorchol-4-ene-3-one (20-HMP) (E); 1(2)-dehydro-22-hydroxy-23,24-bisnorchol-4-ene-3-one (20-HMPD) (F). Figure S2: HPLC chromatograms of standard reference of 20-HMP, 20-HMPD, AD, T, ADD, BD (A) and phytosterol (10 g/L) bioconversion samples (96 h) of *Mycolicibacterium neoaurum* VKM Ac-1815D (B) and *M. neoaurum* VKM Ac-1816D (C). Figure S3: HPLC chromatograms of standard reference of 20-HMP, 20-HMPD, AD, T, ADD, BD (A) and bioconversion samples of AD (4.95 g/L (17.3 mM)) (B) or ADD (4.9 g/L (17.25 mM)) (C) transformation by the *Curvularia* sp. VKM F-3040 resting mycelium. Figure S4: MS spectra of the major steroid products formed by *Curvularia* sp. resting mycelium during cascade biotransformations of phytosterol: testosterone (I), 1-dehydrotestosterone (boldenone) (II), 7α-hydroxytestosterone (III). Figure S5: NMR spectra of steroid metabolites formed by *Curvularia* sp. resting mycelium during cascade biotransformations of phytosterol: testosterone (A), boldenone (B), 7α-hydroxytestosterone (7α-OH-TS) (C), 7α-hydroxyboldenone (7α-OH-BD) (D).
