# Supplementary material for: Boldenone and Testosterone Production from Phytosterol via One-Pot Cascade Biotransformations
Source: J Fungi (Basel). 2024 Nov 28;10(12):830. doi: 10.3390/jof10120830 (PMC11677960; doi:10.3390/jof10120830)
Supplement: Supplementary file 1 [file jof-10-00830-s001.zip › jof-3292927-supplementary.pdf]

Journal:

Journal of Fungi

Title:

Boldenone and testosterone production from phytosterol via one-pot cascade biotransformations

Vyacheslav V. Kollerov*, Tatiana A. Timakova, Andrei A. Shutov, Marina V. Donova

Federal Research Center «Pushchino Center for Biological Research» of Russian Academy of Sciences, G.K. Skryabin Institute of Biochemistry and Physiology of Microorganisms, Prospekt Nauki, 5, 142290, Pushchino, Moscow region, Russia

* Corresponding author:
Vyacheslav Kollerov
svkollerov@rambler.ru
Phone: +7-4967-318584; Fax: +7-495-9563370.

Fig. S1

A

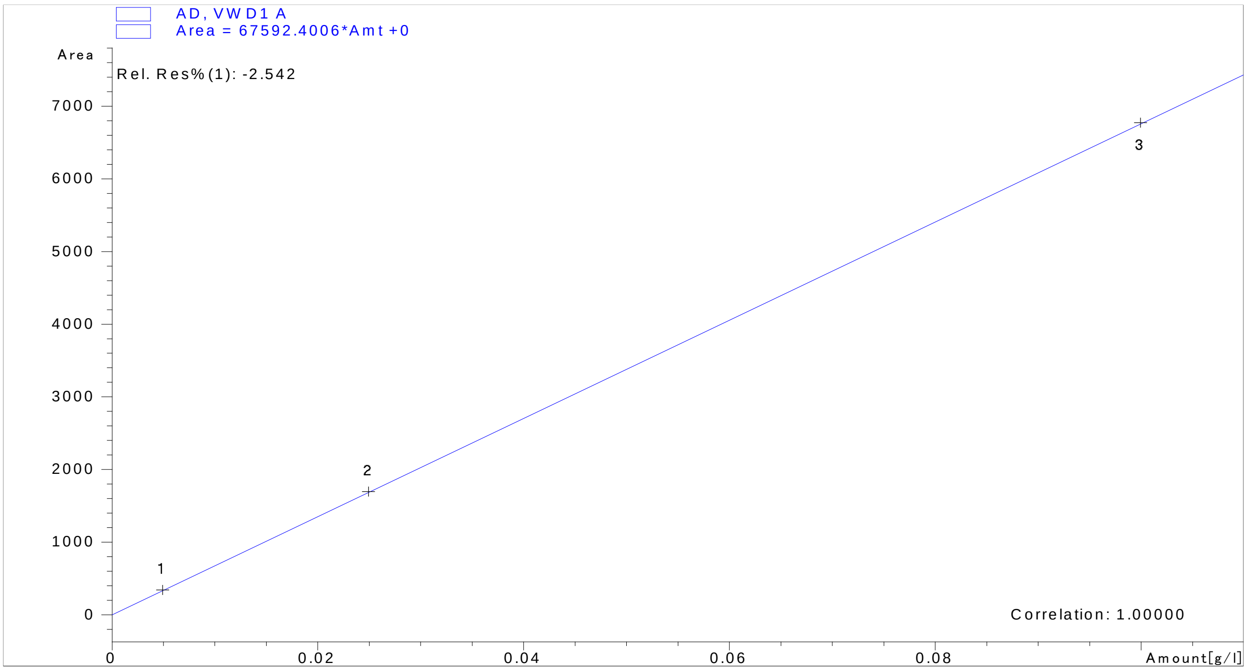
AD, VWD1 A
Area = 67592.4006*Amt +0
Area
Rel. Res%(1): -2.542
7000
6000
5000
4000
3000
2000
1000
0
1
2
3
Correlation: 1.00000
0
0.02
0.04
0.06
0.08
Amount[g/l]

B

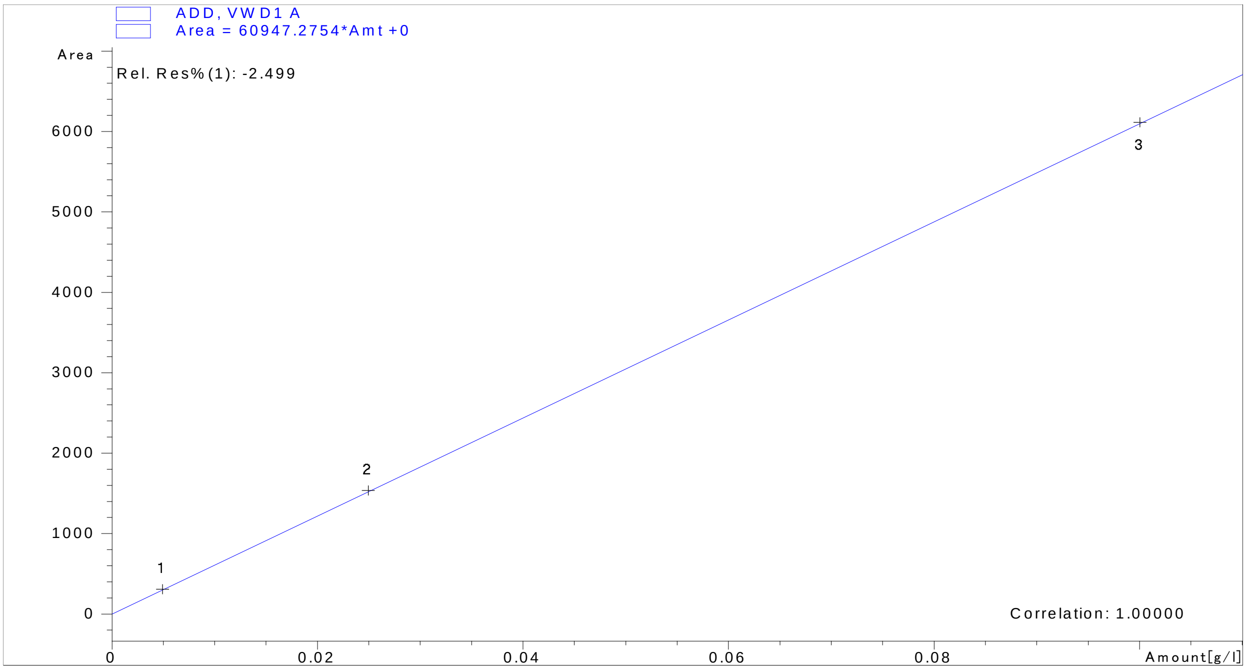
ADD, VWD1 A
Area = 60947.2754*Amt +0
Area
Rel. Res%(1): -2.499
6000
5000
4000
3000
2000
1000
0
1
2
3
Correlation: 1.00000
0
0.02
0.04
0.06
0.08
Amount[g/l]

C

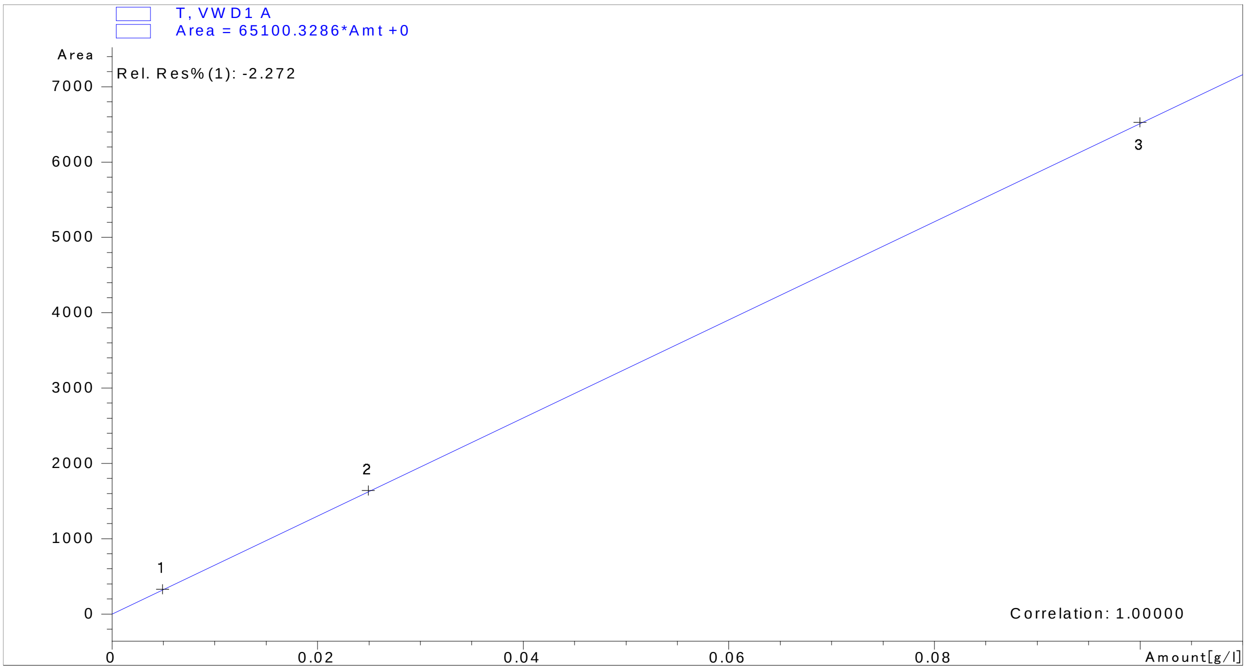
T, VWD1 A
Area = 65100.3286*Amt +0
Area
Rel. Res%(1): -2.272
7000
6000
5000
4000
3000
2000
1000
0
1
2
3
Correlation: 1.00000
0
0.02
0.04
0.06
0.08
Amount[g/l]

D

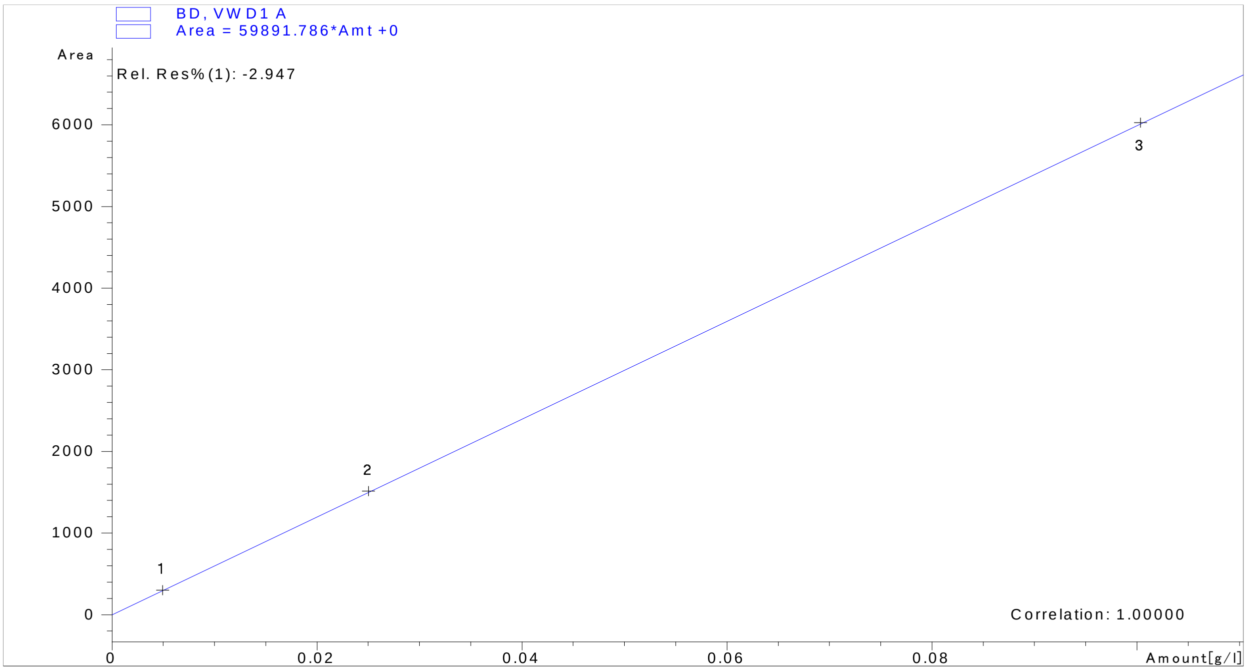
BD, VWD1 A
Area = 59891.786*Amt +0
Area
Rel. Res%(1): -2.947
6000
5000
4000
3000
2000
1000
0
1
2
3
Correlation: 1.00000
0
0.02
0.04
0.06
0.08
Amount[g/l]

E

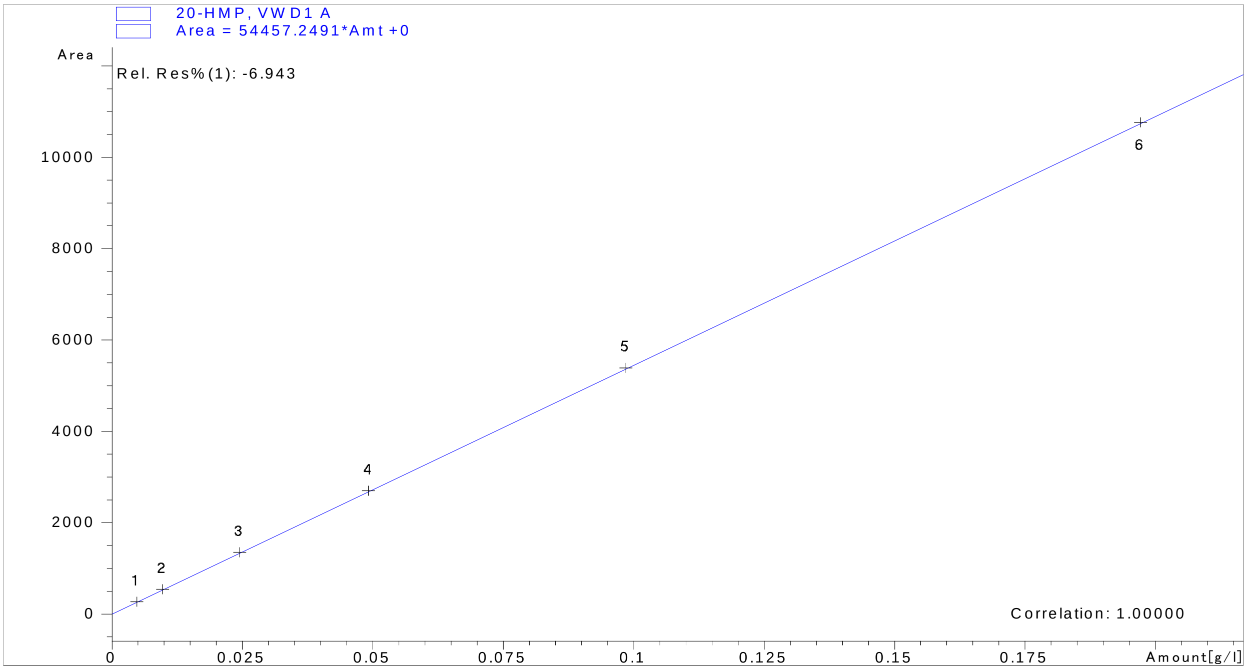

F

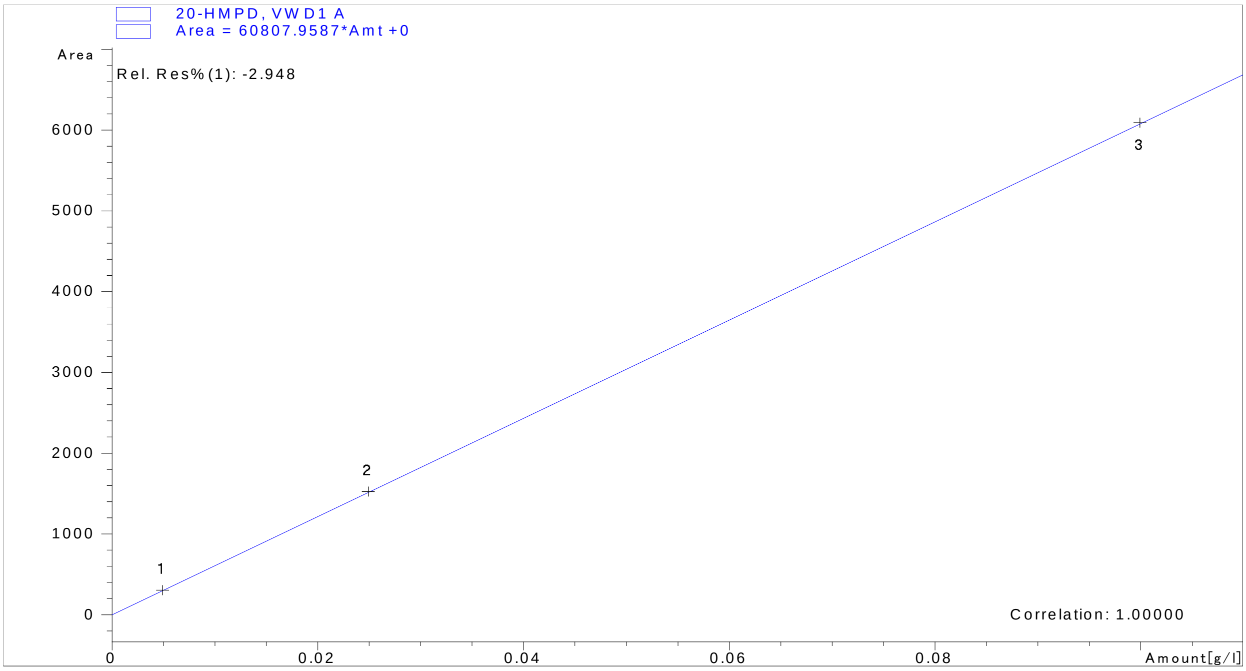

Supplementary Fig. S1. HPLC calibration curves of standard reference of androstenedione (AD) (**A**); androstadienedione (ADD) (**B**); testosterone (T) (**C**), boldenone (BD) (**D**); 22-hydroxy-23,24-bisnorchol-4-ene-3-one (20-HMP) (**E**); 1(2)-dehydro-22-hydroxy-23,24-bisnorchol-4-ene-3-one (20-HMPD) (**F**)

Fig. S2

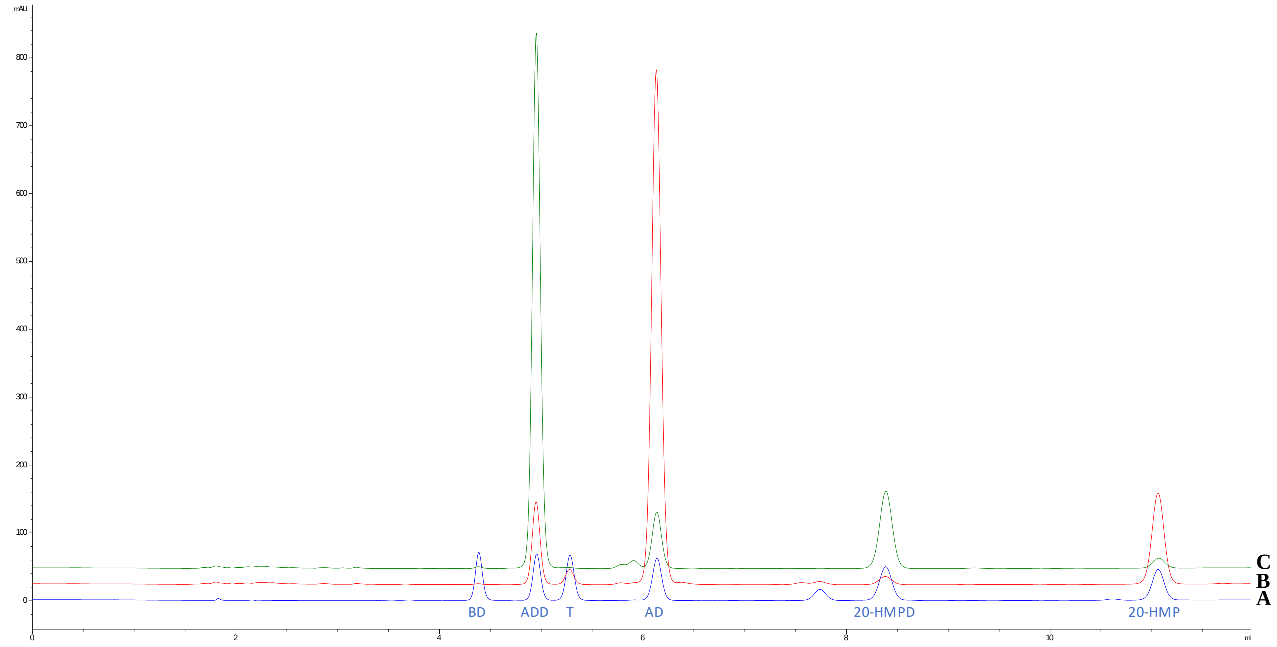

Supplementary Fig. S2. HPLC chromatograms of standard reference of 20-HMP, 20-HMPD, AD, T, ADD, BD (**A**) and phytosterol (10 g/L) bioconversion samples (96 h) of *Mycolicibacterium neoaurum* VKM Ac-1815D (**B**) and *M. neoaurum* VKM Ac-1816D (**C**)

Fig. S3

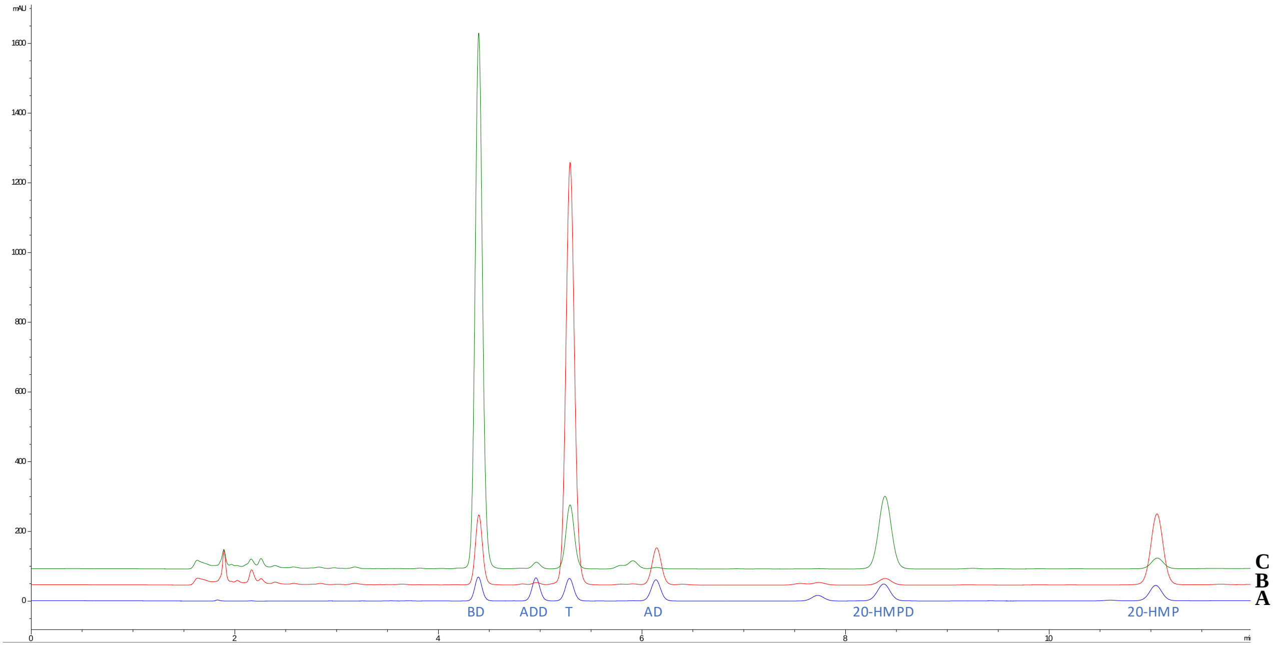

Supplementary Fig. S3. HPLC chromatograms of standard reference of 20-HMP, 20-HMPD, AD, T, ADD, BD (**A**) and bioconversion samples of AD (4.95 g/L (17.3 mM)) (**B**) or ADD (4.9 g/L (17.25 mM)) (**C**) transformation by the *Curvularia* sp. VKM F-3040 resting mycelium

Fig. S4

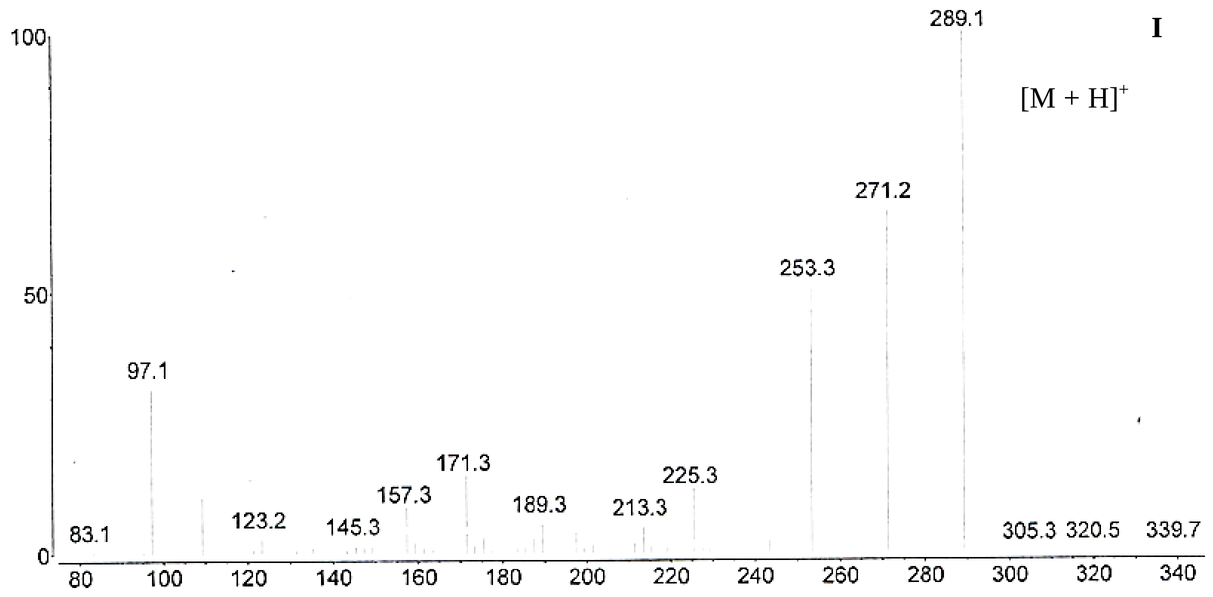

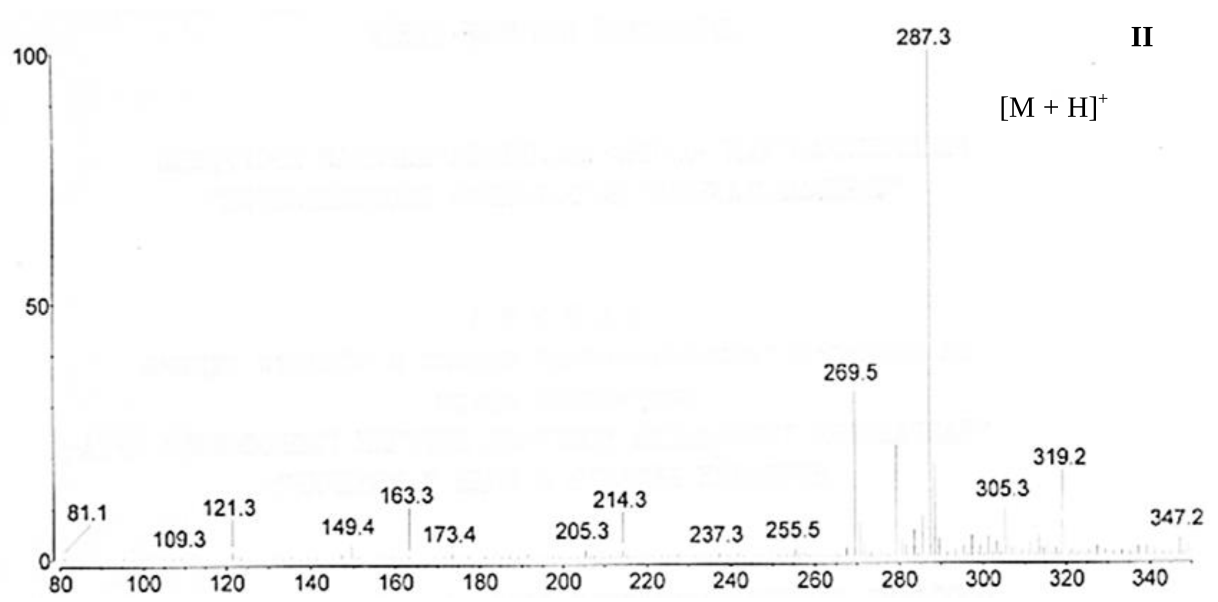

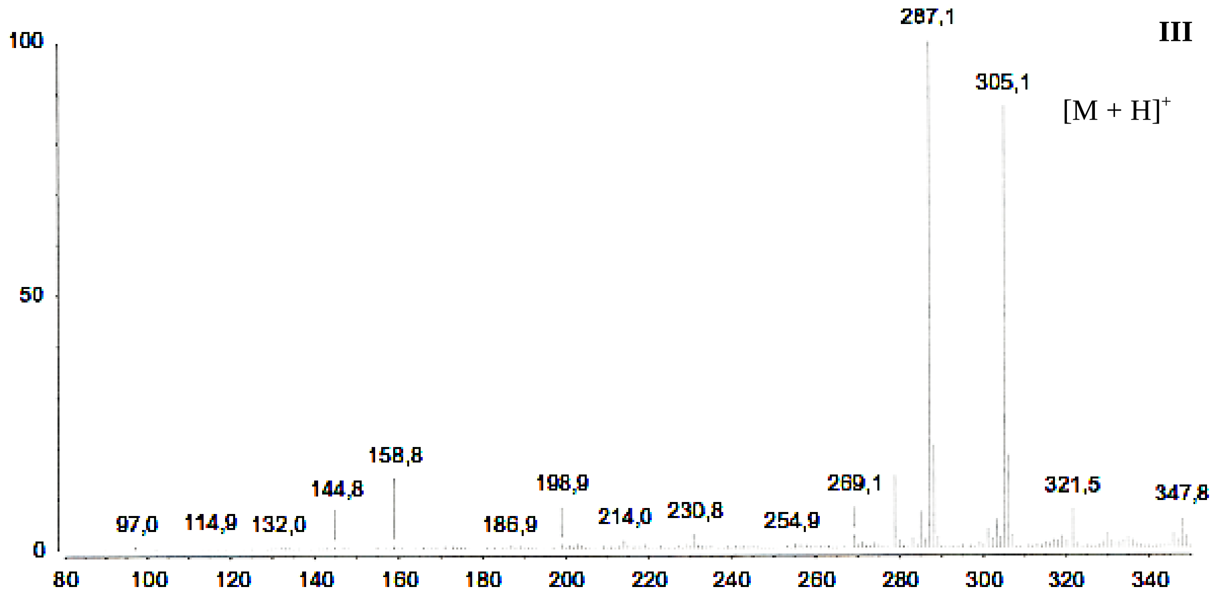

Supplementary Fig. S4. MS spectra of the major steroid products formed by *Curvularia* sp. resting mycelium during cascade biotransformations of phytosterol: testosterone (**I**), 1-dehydrotestosterone (boldenone) (**II**), 7α-hydroxytestosterone (**III**)

Fig. S5.

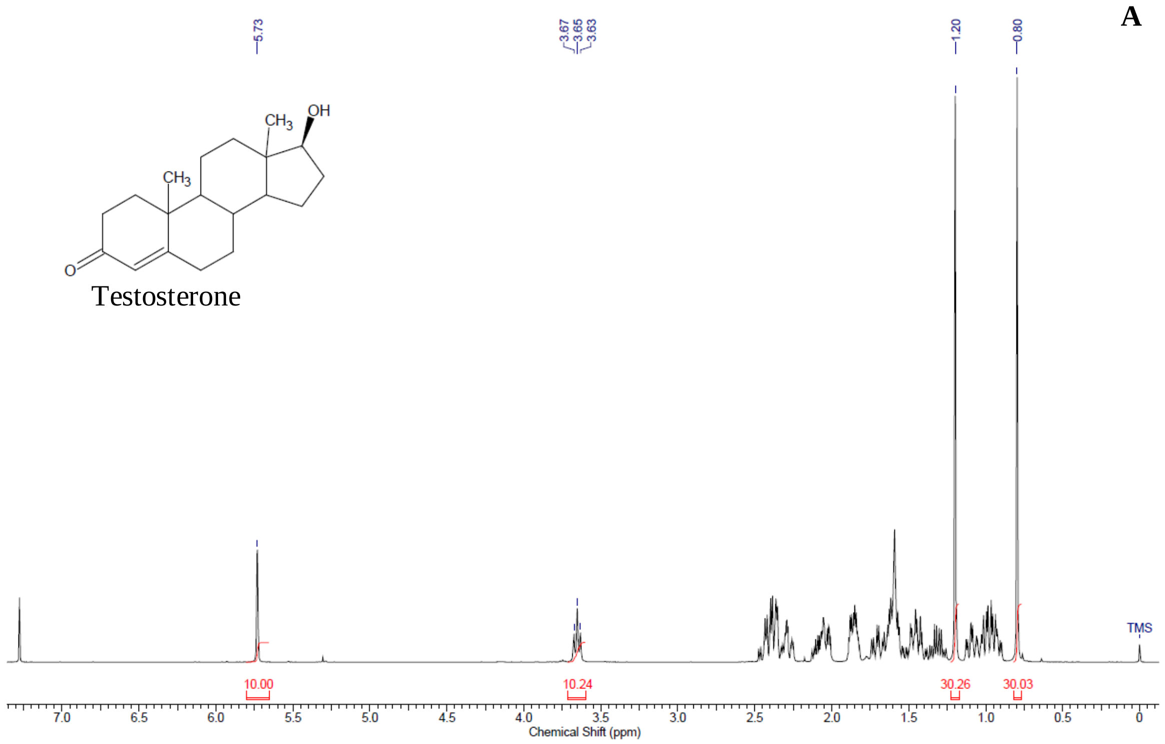

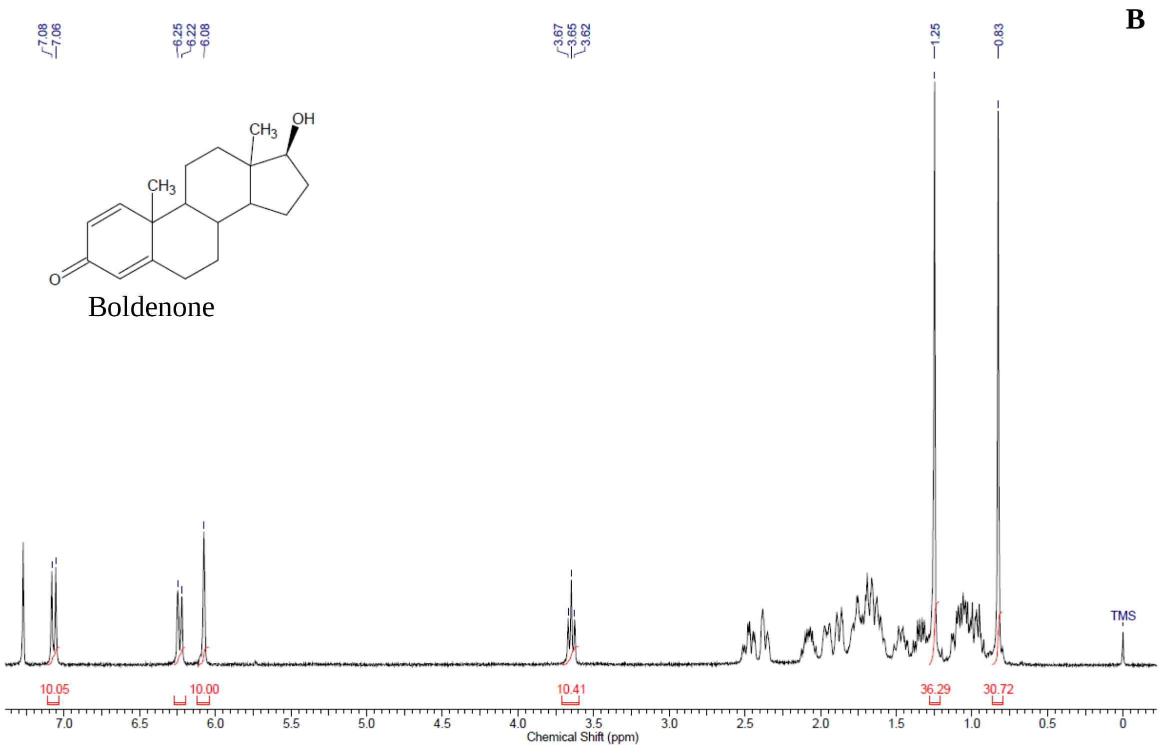
B
7.08
7.06
6.25
6.22
6.08
3.67
3.65
3.62
1.25
0.83
OH
CH3
CH3
O
Boldenone
TMS
10.05
10.00
10.41
36.29
30.72
Chemical Shift (ppm)

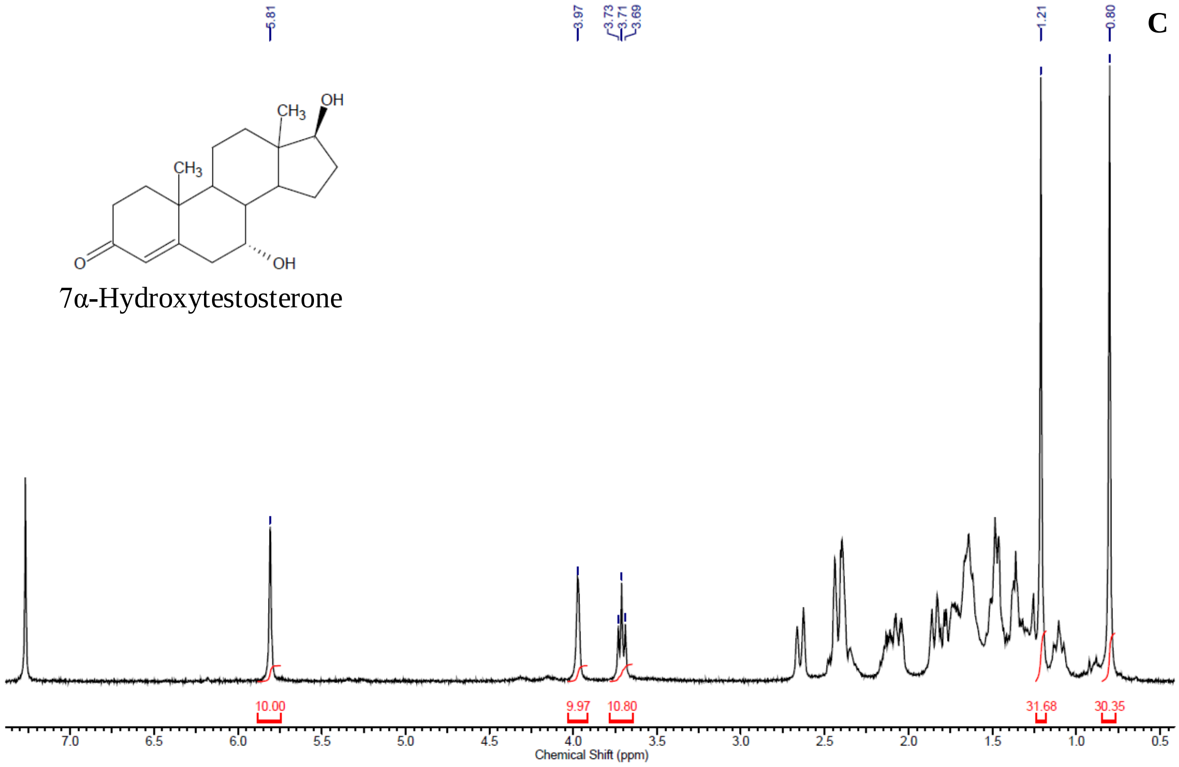
C
5.81
3.97
3.73
3.71
3.69
1.21
0.80
OH
CH3
CH3
O
OH
7α-Hydroxytestosterone
10.00
9.97
10.80
31.68
30.35
Chemical Shift (ppm)

D

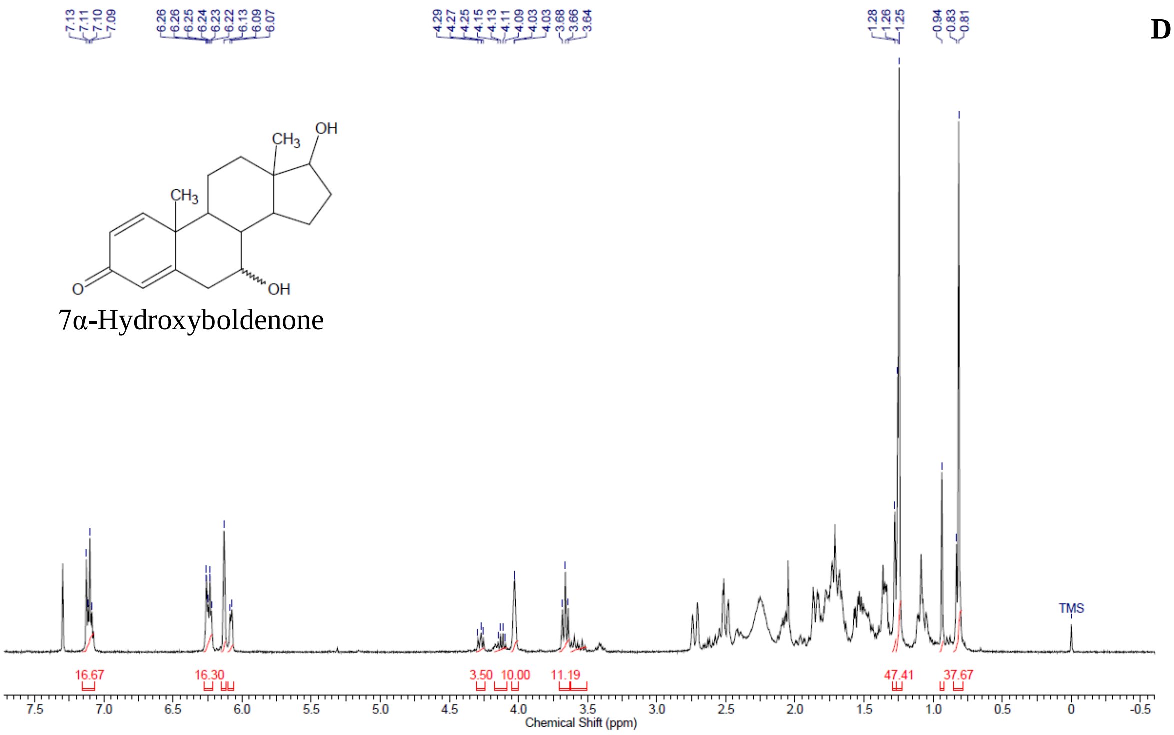

Supplementary Fig. S5. NMR spectra of steroid metabolites formed by *Curvularia* sp. resting mycelium during cascade biotransformations of phytosterol: testosterone (**A**), boldenone (**B**), 7α-hydroxytestosterone (7α-OH-TS) (**C**), 7α-hydroxyboldenone (7α-OH-BD) (**D**)
